# Probing the Origin of the Open Circuit Voltage in Perovskite Quantum Dot Photovoltaics

**DOI:** 10.1021/acsnano.1c05642

**Published:** 2021-12-03

**Authors:** Brian M. Wieliczka, José A. Márquez, Alexandra M. Bothwell, Qian Zhao, Taylor Moot, Kaitlyn T. VanSant, Andrew J. Ferguson, Thomas Unold, Darius Kuciauskas, Joseph M. Luther

**Affiliations:** † National Renewable Energy Laboratory, Golden, Colorado 80401, United States; ‡ Department of Structure and Dynamics of Energy Materials, Helmholtz-Zentrum-Berlin für Materialien und Energie GmbH, Hahn-Meitner Platz 1, 14109 Berlin, Germany; § NASA Glenn Research Center, Cleveland, Ohio, 44135, United States

**Keywords:** perovskite quantum dot, solar cell, open circuit voltage, quasi-Fermi level splitting, electronic traps, time-resolved photoluminescence

## Abstract

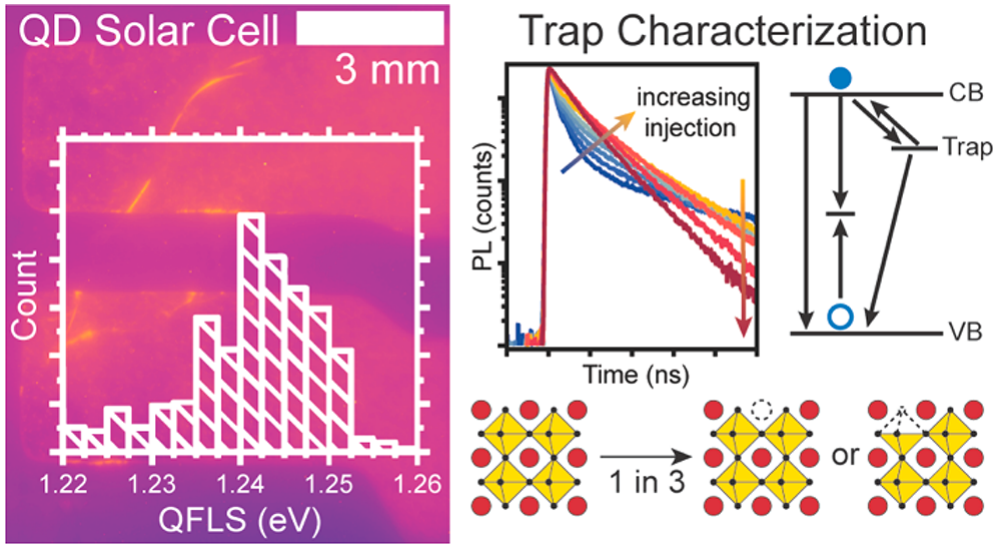

Perovskite quantum dots (PQDs) have many properties that make them attractive for optoelectronic applications, including expanded compositional tunability and crystallographic stabilization. While they have not achieved the same photovoltaic (PV) efficiencies of top-performing perovskite thin films, they do reproducibly show high open circuit voltage (*V*_OC_) in comparison. Further understanding of the *V*_OC_ attainable in PQDs as a function of surface passivation, contact layers, and PQD composition will further progress the field and may lend useful lessons for non-QD perovskite solar cells. Here, we use photoluminescence-based spectroscopic techniques to understand and identify the governing physics of the *V*_OC_ in CsPbI_3_ PQDs. In particular, we probe the effect of the ligand exchange and contact interfaces on the *V*_OC_ and free charge carrier concentration. The free charge carrier concentration is orders of magnitude higher than in typical perovskite thin films and could be tunable through ligand chemistry. Tuning the PQD A-site cation composition *via* replacement of Cs^+^ with FA^+^ maintains the background carrier concentration but reduces the trap density by up to a factor of 40, reducing the *V*_OC_ deficit. These results dictate how to improve PQD optoelectronic properties and PV device performance and explain the reduced interfacial recombination observed by coupling PQDs with thin-film perovskites for a hybrid absorber layer.

Perovskite materials are being explored in a variety of dimensionalities including large single crystal, thin film, nanoplatelet, nanowire, and quantum dot (QD) forms. Each of these morphologies creates opportunities for the development of electronic device technologies and the understanding of fundamental material properties. Perovskite quantum dots (PQDs) have a variety of advantages over perovskite thin films, including (i) stabilization of unstable crystallographic phases through surface strain,^1^ (ii) synthesis of the full spectrum of Cs_*x*_FA_1–*x*_PbI_3_ alloys,^2,3^ (iii) sequential deposition of varying PQD compositions during device fabrication,^4^ (iv) high photoluminescence quantum yields (PLQYs),^5−9^ and (v) low open circuit voltage (*V*_OC_) losses in PQD solar cells compared to thin films of similar compositions.^10^

Building on these advantages, PQD solar cells have demonstrated power conversion efficiencies (PCEs) of up to 16.6% of the AM1.5G solar spectrum.^11,12^ Though a QD solar cell record of 18.1% has been reported,^11^ the composition of this absorber layer is currently unknown. Even with this record, the performance of PQD solar cells lags behind their bulk counterparts, despite the fact that their *V*_OC_ often exceeds that of bulk perovskite solar cells with similar compositions, band gaps, and device architectures, particularly at high band gaps.^10,13,14^ A variety of strategies have now been reported to reduce nonradiative recombination, leading to dramatically improved *V*_OC_’s in absolute and relative (to the thermodynamic limit) terms,^14^ including passivation of the perovskite thin film at the charge transport layer and/or grain boundaries^15−23^ and optimization of crystallization conditions.^24,25^ However, these efforts have focused on lower band gap perovskite thin films suitable for single junction solar cells, not on the higher band gap materials suitable for top junction of tandem solar cells. Since PQD solar cells have not relied on these strategies, the rationale for the high *V*_OC_ in PQD solar cells has remained a mystery. In this study, we develop a fundamental understanding of the relationship between the PLQY, *V*_OC_, and PQD chemistry in solar cells. The absolute PLQY can be used to determine the quasi-Fermi level splitting (QFLS) or the maximum electronic potential the PQDs can provide in a solar cell.

Contactless photoluminescence (PL) techniques can quantify the losses that limit the QFLS and *V*_OC_(26−28) and can identify whether they originate in the PQD film itself or if the loss is associated with an interface of a particular charge transporting layer. For example, through absolute PL measurements, Stolterfoht *et al*. found that the *V*_OC_ from a triple cation bulk perovskite absorber layer with a band gap of 1.62 eV (with a maximum QFLS, *V*_oc_^rad^, of 1.345 V) was limited to 1.121 V due to nonradiative recombination at the perovskite/contact layer interfaces.^29^ This understanding of nonradiative loss mechanisms in perovskite thin film solar cells led to the development of strategies to passivate surfaces, reducing *V*_OC_ losses.^15−23,25,30^

We aim to similarly direct the field of PQD solar cells using various spectroscopic tools to understand the source of *V*_OC_ losses. We complemented absolute PL measurements with time-resolved photoluminescence (TRPL), which can identify the presence and density of defect trap states, both within the absorber and at the absorber/transport layer interface.^31^

These contactless spectroscopic techniques are suited to study the optoelectronic quality with PQD processing, which changes the PQD ligand chemistry. The PQD ligand exchange is an integral part of the fabrication of optoelectronic devices that needs to be considered in loss analysis. To make functional solar cells, PQDs typically undergo two solution-state washing steps as well as a solid-state ligand exchange process to replace the ligands with more compact surface passivants.^32,33^ The high surface area of the PQDs, combined with the susceptibility of ligand desorption during isolation and washing, significantly impacts the optoelectronic properties of the PQD morphology *versus* the bulk films.^33−36^

In this work, we use PL-based measurements to understand *V*_OC_ losses, focusing initially on CsPbI_3_ PQDs. In contrast to thin-film perovskites, both absolute PL and TRPL measurements indicate that the interfaces between the PQD absorber and the charge transport layers have no impact on recombination losses. Rather, the ligand exchange processes required to produce PQD films with good charge transport for high-performance devices result in a 300-fold reduction in the luminescence efficiency relative to the well-passivated, as-prepared PQD films. TRPL measurements indicate that the ligand exchange yields films with (1) a high background carrier concentration not often seen in bulk perovskites and (2) electronic traps that reside *ca*. 150 meV below the conduction band. The high background carrier concentration partially explains why a relatively high *V*_OC_ can be achieved in PQD devices despite the short carrier lifetimes. Replacing the Cs^+^ A-site cation with FA^+^ results in a reduction in the density of electronic traps while maintaining the background carrier concentration, which explains why FA-containing PQDs achieve *V*_OC_’s closer to the detailed balance limit. Finally, we present a model of how the PQD composition alters the surface chemistry, leading to varying trap formation. Our observations indicate that additional attention to PQD surface chemistry is an integral strategy to realize improvements in the PLQY and further enhancement of the *V*_OC_. They also suggest that it may be possible to intentionally tune the charge carrier concentration by controlling the PQD surface passivation, an advantage compared to the bulk perovskite morphology. These studies also illustrate the power of PL-based spectroscopic methods to elucidate the relationship between the PQD surface passivation and the optoelectronic properties, which will strengthen our ability to rationally develop improved PQD optoelectronic devices such as solar cells, detectors, scintillators, and LEDs.

## Results and Discussion

### Impact of QD Processing on the Optoelectronic Properties

One distinction between thin-film perovskites and colloidally synthesized PQDs is the high degree of surface/grain passivation provided by ligands in PQDs. Directly after synthesis, PQDs show extremely high PLQY and are actively explored for their emission properties, but the long native ligands prevent charge transport in PQD films. To promote charge transport, the long-capping ligands must be removed or replaced through ligand exchange using a lead nitrate/methyl acetate (Pb(NO_3_)_2_/MeOAc) solution, reducing the PLQY.^32,33^ Thus, while well-passivated PQDs are touted to show ∼100% PLQY,^5,6,8,37^ the PQD films used in solar cells measured here have film PLQYs <0.1%.

**Figure 1:**
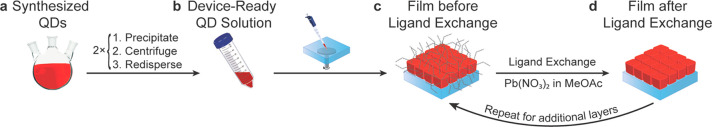
Preparation of CsPbI_3_ QDs and films. (a) As-synthesized QDs were isolated from excess precursors by precipitation and centrifugation to prepare (b) device-ready QDs. The device-ready QDs were deposited *via* spin-coating to form (c) films with a high density of long-chain ligands. Ligand exchange with Pb(NO_3_)_2_ in MeOAc formed a (d) film of device-ready QDs with increased charge carrier mobility.

To start, we employ absolute PLQY measurements to estimate the QFLS, or potential *V*_OC_, of CsPbI_3_ PQDs at varying stages in the processing and device fabrication (Figure 1a–d), including where direct measurement of the *V*_OC_ is not possible due to issues fabricating a functional solar cell. This is particularly useful for quantifying losses in PQDs with a high ligand density in the early processing stages and for probing the impact of the absorber/transport layer interfaces.

**Figure 2:**
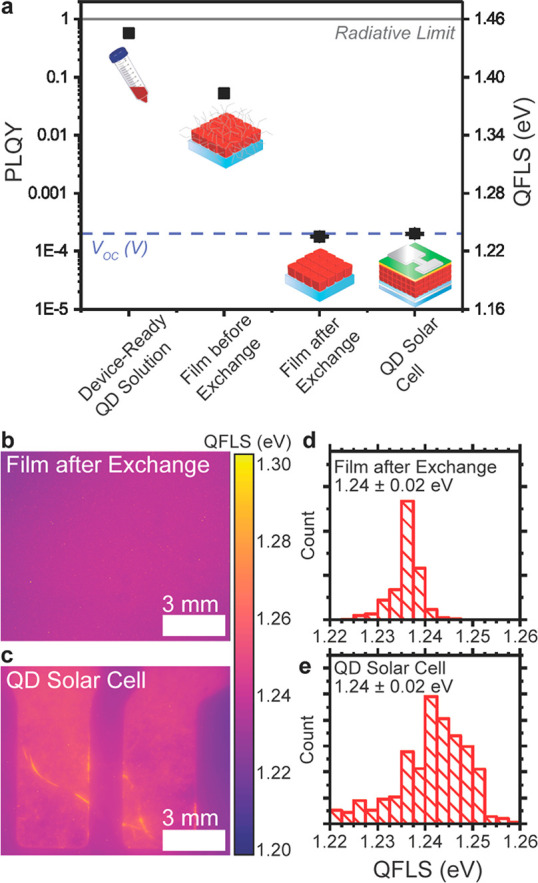
(a) PLQY and the corresponding magnitude of the QFLS of CsPbI_3_ QDs at varying stages in the processing and device fabrication. Spatially resolved absolute PL converted to QFLS of CsPbI_3_ QDs as a (b) film on a bare glass substrate and in a (c) QD solar cell with corresponding QFLS histograms (d and e).

After the PQDs were synthesized^2,32^ (Figure 1a), they were washed in the solution state twice through flocculation and centrifugation, resulting in device-ready PQDs (Figure 1b) primed for deposition. Initially, PQDs in solution (Figure 1a,b) or spin-cast in a film (Figure 1c) have a high density of long, insulating ligands, resulting in a high PLQY (57% in solution and 5.3% in a film, Figure 2a). This high radiative efficiency indicates a high potential *V*_OC_ near the radiative limit of ∼1.46 V, albeit unattainable due to poor charge transport properties.^38^ After solid state ligand exchange of the one-layer-thick PQD film to improve charge transport, the PLQY drops significantly, from 5.3% to ∼0.02%, corresponding to QFLS values of around ∼1.24 V measured at 1 sun illumination levels. This value sets the upper limit for the *V*_OC_ of PQD films after ligand exchange and is in near total agreement with the *V*_OC_ measured in PQDs solar cells (Figure S1).^10^ This indicates that the internal voltage generated in the absorber layer at open circuit is collected externally in a full device, suggesting that the contacts do not reduce the *V*_OC_ (in contrast to thin-film devices) and that the ligand exchange process is the primary origin of voltage losses in our CsPbI_3_ PQD devices.

### Insensitivity of the Optoelectronic Properties to Contact Interfaces

To further prove this point, we then compared the absolute PL under 1 sun conditions for the absorber layer on glass (Figure 2b,d) and a full PQD device (Figure 2c,e) with the architecture ITO/TiO_2_/PQD/Spiro/MoO_*x*_/Al. The experimental details for solar cell fabrication as well as a representative *J*–*V* curve of a PQD solar cell are included in the Methods and Experimental Details section and Supporting Information. Localized increase of the PL yield is observed as a result of reflections caused by the metal electrodes, which are visible in the QFLS map of the PQD solar cell. As expected, there is no significant change in the QFLS of the QDs with and without contacts, where the standalone QD film had a QFLS of 1.24 ± 0.02 eV, while the QFLS of the QDs in the PQD solar cell was 1.24 ± 0.02 eV, where the experimental error is instrument-limited to 0.02 eV.

By monitoring the PLQY at each processing stage (Figure 2a), we find that the ligand exchange causes a significant loss in the QFLS, which remains constant when the PQDs are in contact with either the ETL or HTL, revealing that the voltage losses in CsPbI_3_ solar cells originate from the PQD absorber layers themselves and not from the contacts. Absorption and PL spectra were used to ensure no significant changes in the band gap before or after ligand exchange for one- or four-layer-thick PQD films (Figure S2). Furthermore, testing a PQD solar cell that was encapsulated in an inert atmosphere showed no difference in the QFLS, indicating that the voltage losses are not a product of degradation, but instead are inherent to the as-prepared PQD film (Table S1). Since the maximum open circuit voltage, *V*_oc_^rad^ (equal to the maximum quasi-Fermi level splitting in the radiative limit) in our films is ∼1.46 V (see Figure S3 for details), the nonradiative recombination voltage loss in our CsPbI_3_ PQD solar cells is ∼220 mV, or about 15% of the *V*_oc_^rad^. Some thin-film bulk perovskite solar cells with smaller band gaps have reduced nonradiative bulk recombination.^20,22^ For example, the passivation of the FA_1–*x*_MA_*x*_PbI_3_ perovskite surface using phenethylammonium iodide reduced voltage losses to as low as 70 meV, or 5.6% of the *V*_oc_^rad^.^22^ This is perhaps unsurprising, since the dramatically increased surface area within PQD films will increase the density of defects associated with the PQD surfaces, pointing toward opportunities to modify the PQD processing and optimize the surface chemistry to retain a high PLQY and device *V*_OC_.

These results will aid the development of wide band gap perovskites for use in tandem photovoltaics, which require a higher band gap than these single junction thin-film devices. Nevertheless, the device *V*_OC_ for perovskites in both bulk and QD morphologies approaches a plateau as the band gap increases from 1.7 to 2.3 eV,^10^ suggesting an increase in *V*_OC_ losses with band gap.^39^ The *V*_OC_ of bulk perovskite solar cells is typically limited by the interfaces between the optically active perovskite layer and the electron and hole transport layers (ETL and HTL, respectively).^29,40−42^ In contrast, the data described above suggest that the interfaces with the contact layers do not introduce significant voltage losses in our CsPbI_3_ PQD solar cells with a band gap of 1.746 eV. This is consistent with previous results using Kelvin probe force microscopy, which showed that PQDs reduce deleterious interfacial recombination.^43^

### Time Resolved Photoluminescence of CsPbI_3_ QD Films

**Figure 3:**
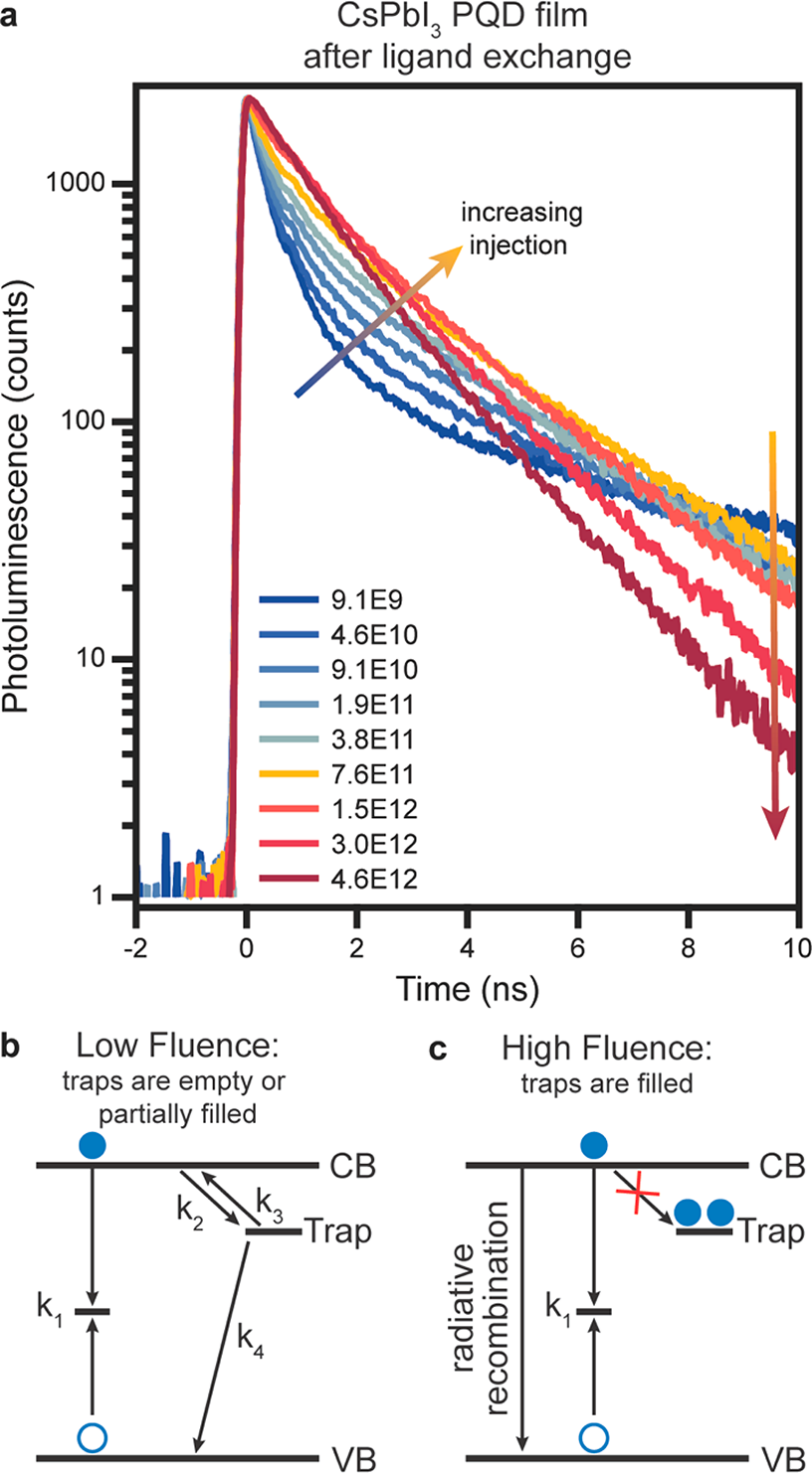
(a) TRPL data for CsPbI_3_ PQD films after ligand exchange with varying 470 nm excitation photon fluence (photons/(pulse·cm^2^)) indicated in the legend. Schematic indicating carrier dynamics at (b) low fluence and (c) high fluence.

After using PLQY measurements to establish that the ETL and HTL interfaces do not limit the voltage, we turn to time-resolved studies to gain insight into the traps that are postulated to form during the ligand exchange process, reducing the PLQY. Using TRPL, we varied the excitation fluence to reveal the presence and density of carrier traps and apply kinetic models to determine the trap electronic properties^31^ and the density of background charge carriers in the PQD films.^44^ The TRPL decay data at varying excitation fluences are plotted in Figure 3a for the CsPbI_3_ PQD film after ligand exchange, revealing a transition from low charge carrier injection (blue traces) to high charge carrier injection (orange and red traces).

First, in the low injection regime, the influence of trap states in the CsPbI_3_ PQDs can be directly observed, which manifests as nonexponential behavior with a fast decay component due to the capture of carriers in the traps. The PL lifetimes change as a result of the charge carrier trapping with changing excitation fluence. The increase in excitation fluence from 9.1 × 10^9^ photons cm^–2^ pulse^–1^ to 7.6 × 10^11^ photons cm^–2^ pulse^–1^ (Figure 3a, blue to yellow traces) leads to a reduction in amplitude and increase in decay time for the fast decay component (Figure 3a, blue to yellow arrow), indicating saturation of the minority charge carrier traps. Upon saturation, the lifetimes in the intermediate injection regime (yellow TRPL trace) indicate defect-mediated recombination.

The second change in the TRPL data occurs in the high injection regime when the trap state is filled (Figure 3a, yellow to red traces) and the defect-mediated recombination (*k*_1_) dominates the TRPL (Figure 3c). In this regime, the PL flux is proportional to Δ*n*^2^ (where Δ*n* is the excess photogenerated charge carriers), which leads to a reduction in the TRPL decay time from τ (low injection) to τ/2 expected for high injection (Figure 3a, yellow to red arrow; lifetimes tabulated in Table S2), where τ is the defect-mediated recombination lifetime. The CsPbI_3_ PQDs in the ligand-exchanged thin films and solar cells studied in this work have a PLQY of 0.02% (Figure 2a). This means that only 0.02% of all recombination is radiative and 99.98% of recombination is nonradiative, where excess energy is dissipated *via* phonon emission. Nonradiative recombination typically occurs *via* defect states, by an assumed process of electron and hole capture by such states (*k*_1_ in Figure 3b,c). Defect-mediated nonradiative recombination is more probable because it requires emission of a smaller number of phonons than band-to-band nonradiative recombination.

The trap saturation was analyzed using a previously reported kinetic scheme, illustrated in Figure 3b and described in the Supporting Information, to estimate trap densities in device-ready ligand-exchanged PQD films.^31,45^ CsPbI_3_ PQD films have been measured to have p-type background doping, meaning the minority carriers are electrons in the conduction band (CB) and the trap states are localized near the CB.^46^ This model is still valid in the case of an n-type perovskite, but the holes in the valence band (VB) would act as the minority charge carrier and the trap state would be near the VB. In this kinetic model, the trap states are empty prior to photoexcitation, such that when an electron is excited from the VB to the CB, the electron may undergo defect-mediated recombination (*k*_1_) or be trapped (*k*_2_), potentially resulting in an additional channel of defect-mediated recombination (*k*_4_). On the other hand, the trapped electron could be detrapped back to the CB (*k*_3_), allowing for delayed radiative recombination, which results in increased TRPL lifetime measured at the lowest excitation fluence of 9.1 × 10^9^ photons/(cm^2^pulse^–2^). The detrapping and trapping rates are related by , where *E*_a_ is activation energy, *k*_B_ is the Boltzmann constant, *T* is temperature, *N*_t_ is trap density, *N*_C_ is density of states in the conduction band, *n* is electron density in the conduction band, and *n*_t_ is electron density in the trap state.

**Figure 4:**
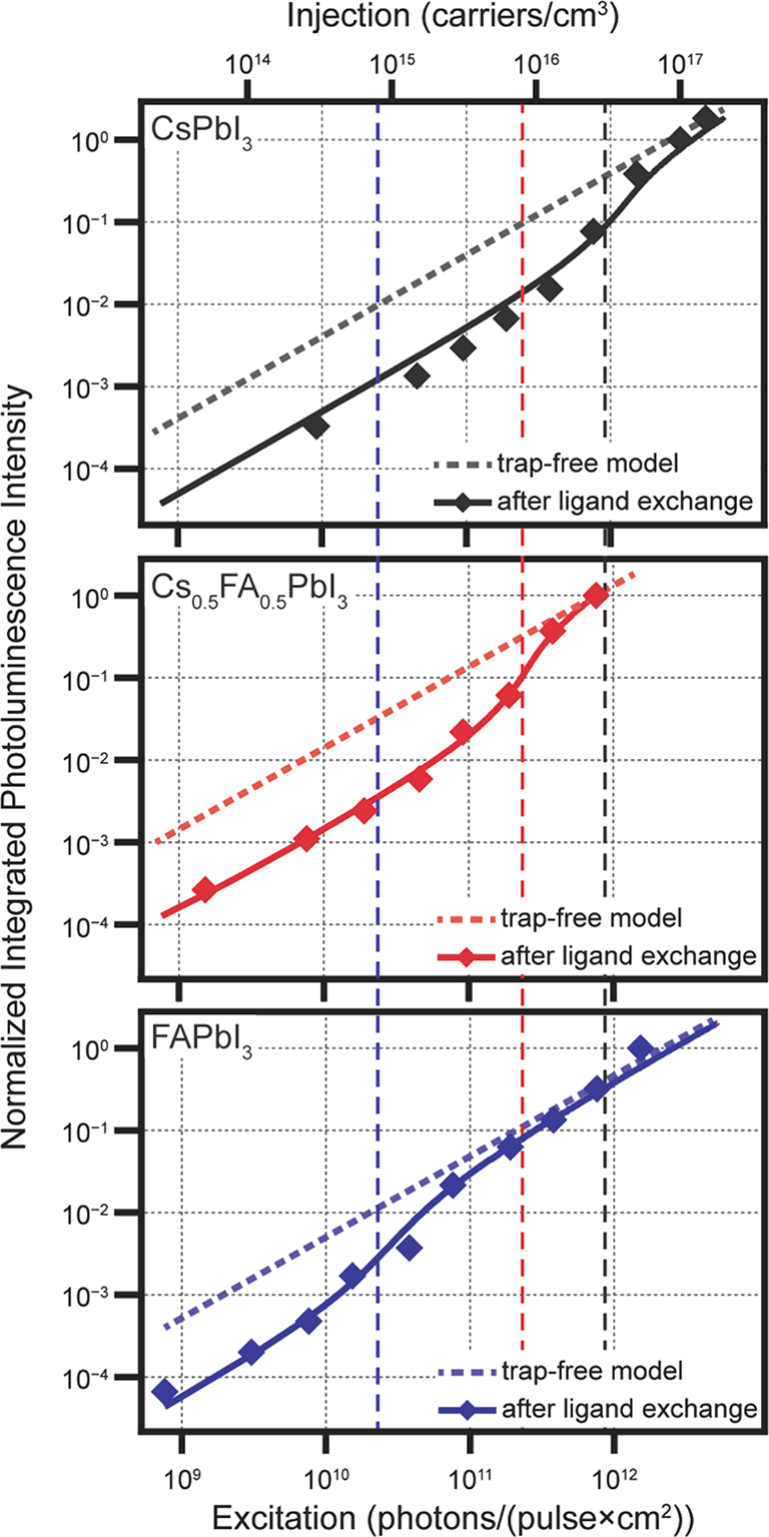
Normalized integrated PL intensity (data points) *versus* excitation fluence with model results (lines) for CsPbI_3_ (black), Cs_0.5_FA_0.5_PbI_3_ (red), and FAPbI_3_ (blue) PQD films. The trap densities derived from the model results are plotted as vertical dashed lines where values correspond to the upper axis.

The normalized time-integrated TRPL data in Figure 4 allow simple visualization of trapping effects in CsPbI_3_ (upper panel). The PL intensity of the PQD film after ligand exchange (black diamonds) has two distinct linear regimes at low and high excitation fluences with an offset in between. At low excitation fluence, carrier trapping causes a reduction in the PL intensity compared to the expected integrated PL intensity for a PQD film without traps (Figure 4, black line). Upon trap saturation at high excitation fluence, the PL intensity again increases linearly at a higher level equal to the trap-free model. The excitation fluence at which the transition between these two regimes occurs allows quantification of the trap density, which can be read from the estimated injection (upper axis in Figure 4) and also numerically modeled, indicating the density of traps is approximately 3 × 10^16^ cm^–3^ (vertical dashed black line, Figure 4). The modeled transients at low injection are plotted in Figure S4, and trap properties are summarized in Table S3. Therefore, the traps in ligand-exchanged CsPbI_3_ PQD films are filled at a fluence of about 10 suns. The magnitude of the PL offset (vertical shift in integrated intensity) depends on the defect-mediated recombination rate *k*_1_ and trap energy *E*_a_. Because *k*_1_ is determined directly from kinetic data (Figure 3), the offset indicates a trap energy *E*_a_ ≈ 200 meV in CsPbI_3_ PQD films. These traps may be responsible for reducing the *V*_OC_ of CsPbI_3_ QD solar cells by approximately 60 mV, inferred from the roughly 1 order of magnitude difference in PL intensity when traps are empty *versus* filled. We also collected TRPL data for CsPbI_3_ PQD films prior to ligand exchange (see Figure S5 and more detailed discussion in Supporting Information) but are unable to use this model to quantify trap properties since charge carrier dynamics in these samples are dominated by radiative recombination.

Based on these absolute PL and TRPL measurements, we reason that traps are introduced during the solid-state Pb(NO_3_)_2_/MeOAc ligand exchange process, in which oleate ligands are exchanged for acetate ligands (Figure 1d),^33^ and further reason that the traps are neither introduced nor passivated by the contact layers (Figure 2). This characteristic is distinctly different from non-QD perovskite films, where increased recombination is often associated with the interfaces between the perovskite absorber and carrier transport layers.^29,40−42^ This further explains why the *V*_OC_ can be improved in some perovskite photovoltaics (PVs) where QDs are inserted between the perovskite thin films and contact layers.^43,47^ This comparison indicates that different device optimization strategies are currently needed for PQDs compared to thin-film perovskites, and PL measurements on PQD films allow rapid evaluation and detailed quantification of the efficiency-limiting defect states.

The TRPL data also allow for the background carrier density in the PQDs to be determined. At fluences >1.5 × 10^12^ photons/(cm^2^ pulse) (Figure 3a), the TRPL lifetimes are reduced, which is attributed to the transition to high injection. This occurs when the photogenerated carrier density is higher than the background carrier density in the absorber. Analysis of this aspect of the data can be used to estimate free charge carrier concentration in the absorber, which is an important characteristic because it determines the majority carrier (here, hole) quasi-Fermi level. To estimate the background carrier concentration, we numerically solved coupled time-dependent differential equations for electrons/holes in the conduction/valence bands and in two defect levels to calculate the radiative recombination rate.^44^ This kinetic model is described in Figure S6 and eqs S4–S10. Simulation results when the free charge carrier concentration is varied while holding all other material parameters constant are shown in Figure S7a. By comparing the experimental data at excitation fluence 4.4 × 10^12^ photons/(cm^2^ pulse), we find that the free charge carrier concentration *p* ≈ 2 × 10^18^ cm^–3^ for CsPbI_3_ PQD films after the solid-state ligand exchange. The estimate has large uncertainty, because it relies on values of charge carrier capture, emission cross sections, and other parameters that could be injection dependent and can vary spatially in the films. Therefore, we do not conclude that the background charge carrier concentrations are significantly different for varying PQD film composition (simulations shown in Figure S7). In all cases, the value of *p* is orders of magnitude larger than in bulk perovskite films studied previously and is likely the result of the large number of crystallites in PQD films.^48−50^ With a density of QDs of ∼2 × 10^17^ cm^–3^, each QD must have an average of 10 free charge carriers to have such a high background carrier concentration. In contrast, the crystallites in a bulk perovskite thin film can be orders of magnitude larger in every dimension, increasing the number of free charge carriers per grain necessary to match the same free charge carrier concentration. Compared to other QD compositions, this free charge carrier concentration is comparable to that found in lead chalcogenide QDs, 10^18^ to 10^19^/cm^3^.^51^

The background carrier density is an important factor in the overall device performance, particularly in the presence of electronic traps. A high background carrier density has been shown to improve the *V*_OC_ in CdTe by filling traps that lead to nonradiative recombination, thereby increasing the PLQY and *V*_OC_.^52^ The PLQY increases for larger QFLS, where (for p-type absorbers) the electron quasi-Fermi level depends on the minority carrier lifetime and the hole quasi-Fermi level depends on free charge carriers. PQDs have high background carrier density resulting in *V*_OC_’s that can exceed thin-film perovskite *V*_OC_’s for comparable recombination lifetimes. This suggests that by controlling the PQD surface passivation, it may be possible to intentionally tune charge carrier concentration, an ability that may be unique to PQD films compared to bulk perovskites. Similar strategies of using charged ligands to modify surface charge to increase PV performance are also used for thin-film perovskites,^53−58^ but the much larger interface areas for PQDs could make this approach more efficient and tunable.

### Effect of Varying A-Site Composition on Charge Carrier Dynamics

Cs_*x*_FA_1–*x*_PbI_3_ PQD alloys have attracted significant attention due to the reduced voltage deficit observed.^2,10,12^ Based on our observation that the ligand exchange process controls the background carrier and surface trap densities, directly impacting the QFLS (and hence *V*_OC_), we reason that these improved optoelectronic properties may arise from a modulation of the PQD electronic properties. We found that the trap density significantly decreases with increasing FA content but that the background charge carrier concentration is similarly high across all compositions of PQDs.

The TRPL decays of Cs_0.5_FA_0.5_PbI_3_ and FAPbI_3_ with varying excitation fluence (plotted in Figure S8) were similarly used to investigate the trap density (low injection regime, blue traces) and charge carrier concentration (high injection regime, red traces). The first observation from the TRPL decay data is that the transition from the low to high injection regime occurs at approximately the same excitation fluence (≥1.5 × 10^12^ photons cm^–2^ pulse^–1^) irrespective of the FA content (yellow trace). This indicates that replacing Cs^+^ with FA^+^ does not alter the chemistry that results in free charge carriers, suggesting that the free charge carriers may result from either iodide or surface ligand vacancies. In contrast, trapping/detrapping dynamics evident in the low injection regime (blue to yellow traces) are very different with A = Cs^+^/Cs_0.5_FA_0.5_^+^/FA^+^ cations, indicating that traps are changed by A-site modification.

The time-integrated TRPL data as a function of excitation fluence immediately reveal several differences with varying A-site composition (Figure 4, middle and lower panels). In particular, the transition between the two linear regimes, which determines the trap density, is reduced with increasing FA content. These integrated TRPL data indicate a *ca*. 4× reduction in trap density (8 × 10^15^ cm^–3^) in the Cs_0.5_FA_0.5_PbI_3_ PQDs (Figure 4, vertical dashed red line) compared to the CsPbI_3_ PQDs. The trap density is further reduced by a factor of 10× in the FAPbI_3_ PQDs to *ca*. 8 × 10^14^ cm^–3^ (Figure 4, vertical dashed blue line). The reduced trap density is immediately apparent by comparing the vertical dashed lines, indicating trap density, which decrease with increasing FA content. Additionally, the smaller offset between the two linear regimes possibly points to a reduction in the trap depth (*E*_a_) in addition to the reduced trap density. Table S3 summarizes trap properties for CsPbI_3_, Cs_0.5_FA_0.5_PbI_3_, and FAPbI_3_ PQD films after ligand exchange. This difference in trap density is further corroborated by PLQY measurements of PQDs with varying composition (Figure S9) in solution and in a film before and after ligand exchange. The PLQY of all QD compositions is high in solution and drops upon deposition as a film and after ligand exchange, but the decrease in PLQY after ligand exchange is directly proportional to the Cs content of the PQDs.

### Discussion of the Nature of Trap States and Free Charge Carriers

These comparisons allow us to draw several conclusions that improve our understanding of PQD solar cells and PQD optoelectronics. First, by comparing the trap density of PQDs with different A-site compositions, we infer that A-site composition directly affects the trap formation. Furthermore, since the background carrier concentration is similar for all compositions, the free charges and traps in our samples are created by different chemical properties, and variation of the A-site composition can be used to reduce trap density while maintaining the high background charge carrier concentration. The alloyed Cs_0.5_FA_0.5_PbI_3_ PQD films, therefore, do not suffer from the high density of traps formed in the CsPbI_3_ PQD film, but benefit from the same increase in background charge carrier concentration. This helps explain why these PQDs can achieve *V*_OC_’s closer to the radiative limit and yield improved efficiencies, particularly over CsPbI_3_ PQD solar cells.^2,10,12^

Comparing PQD films with different A-site cations provides additional evidence for the trap’s chemical identity and particularly highlights the importance of the A-site composition on the electronic properties of the PQDs. Our results are consistent with an AX facet termination of the PQDs, which was shown both theoretically (using DFT) and experimentally (*via* NMR) in CsPbBr_3_ PQDs.^34,35^ Theoretical studies of CsPbBr_3_ PQDs suggest that various surface species can be lost during the washing process, including CsX′ (where X′ = halide and/or oleate), PbX_2_ after the underlying PbX layer is exposed (where X = halide), and OLAmX (where OLAm = oleylammonium, X = halide).^8,34^

**Figure 5:**
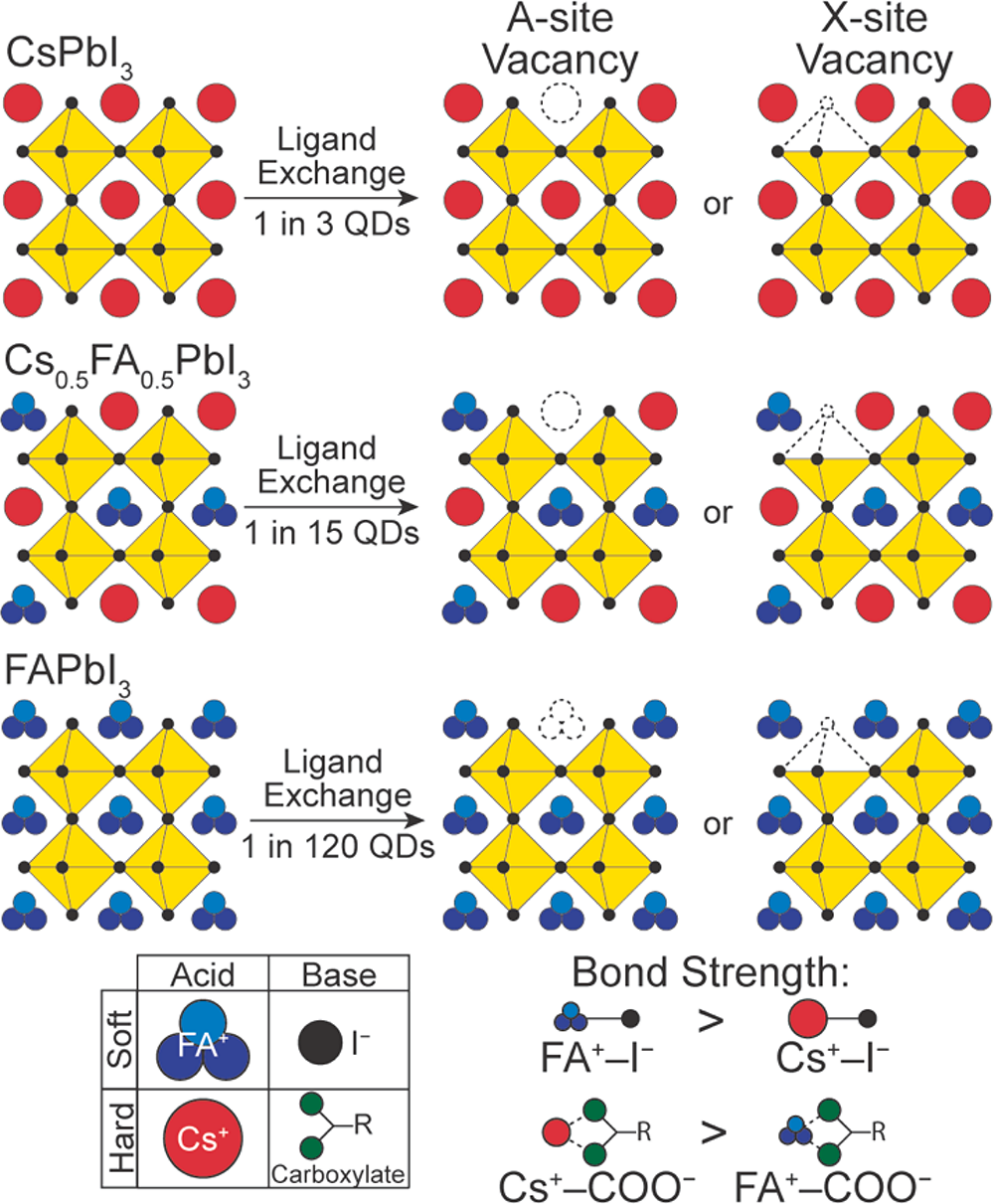
Trap formation in CsPbI_3_, Cs_0.5_FA_0.5_PbI_3_, and FAPbI_3_ QDs, labeled with the average number of QDs with a trap for each QD composition. The relative hardness of formamidinium, iodide, cesium, and carboxylate ions is labeled, leading to the relative bond strengths.

Assuming that the CsPbI_3_ and FAPbI_3_ PQD surfaces are terminated by an AX surface, similar to the CsPbBr_3_ PQDs, we reason that either surface A-site or X-site vacancies are the source of the traps formed during the ligand exchange process (Figure 5). Hard soft acid base (HSAB) theory could explain the chemical interactions that control trap formation during the ligand exchange process. In the case of an A-site vacancy causing the trap site, the defect would be formed by the loss of either cesium or formamidinium carboxylate. Since Cs^+^ and carboxylate ions are relatively hard, desorption of cesium carboxylate during the ligand exchange is more likely than desorption of formamidinium carboxylate, since formamidinium is a softer base and would therefore have a weaker interaction with the hard carboxylate anion. Therefore, the number of A-site vacancies, and hence the density of trap sites, would be greater in CsPbI_3_ than in FAPbI_3_. Alternatively, the A-site composition could impact the formation of iodide vacancies, which could be the chemical identity of the trap sites. Because the interaction between neighboring FA^+^ and I^–^ is relatively strong, since they are both soft ions, the formation of I^–^ vacancies would be reduced in FA-containing PQDs compared to CsPbI_3_ PQDs. The formation of I^–^ vacancies is consistent with previously reported *ab initio* calculations that identify halide vacancies that exhibit trapping behavior.^8^ These hypotheses, however, do not exclude the possibility that more complex surface atomistic structures result in electronic traps, such as the loss of CsI or complete loss of the outer AX shell, exposing the underlying BX layer.^34,59^

Regardless of the precise nature of the trap sites, it is notable that the A-site composition so strongly impacts the electronic properties of the PQDs, and therefore their utility in solar cells and other optoelectronic devices. Typically, the A-site is not considered to contribute to the electronic properties of the perovskites, since the band edge states are primarily based on the lead and halide orbitals.^2,60−65^ These experiments not only highlight the importance of the A-site to the electronic properties of the PQDs but also emphasize the importance of the ligand exchange process to PQD devices. Although the ligand exchange introduces deleterious traps, reducing the overall solar cell performance, the ligand exchange process is necessary to create PQD solar cells. First, the ligand exchange is necessary to deposit multiple layers of the PQDs, allowing nearly complete harvesting of the incident light. Second, by replacing the long-chain oleate ligands with acetate ligands, the PQDs are able to couple to each other electronically, allowing effective charge transport through the film. The extent of this ligand exchange remains an open question in the PQD field; incomplete acetate passivation could affect the PQDs’ optoelectronic properties. Ligand removal without ligand exchange could be responsible for the change in free carrier concentration. These experiments emphasize the importance of developing ligand exchange and surface passivation strategies that enable the deposition of a conductive film of PQDs thick enough to absorb the incident solar irradiation while simultaneously preventing trap formation.

## Conclusions

Absolute PL measurements have been used to assess the losses in PQD solar cells, revealing that the *V*_OC_ is not limited by interface recombination, in contrast to their bulk perovskite counterparts. Instead, the ligand exchange process, necessary to ensure high charge carrier mobility across the PQD film, reduces the PLQY to ∼0.02%. This points to the surface chemistry of the PQDs as the critical locus for carrier recombination losses that limit the QFLS, corresponding to an implied *V*_OC_ of ∼1.24 V compared to the radiative limit of 1.46 V. TRPL measurements have been used to further characterize the PQD films, finding that the ligand exchange process introduces surface traps and significantly increases background free charge carriers in CsPbI_3_ PQDs to levels significantly higher than those achieved in bulk perovskite thin films. The increased background carrier concentration compensates for the reduced carrier lifetime due to bulk and surface traps, which helps to explain the better *V*_OC_ observed in PQD solar cells *versus* their bulk perovskite counterparts. These results also indicate that the free charge carrier concentration could be tuned through careful control of the PQD ligand exchange. Lastly, TRPL measurements of FA-containing PQDs indicate that the ligand exchange process still beneficially increases the background charge carrier concentration of Cs_0.5_FA_0.5_PbI_3_ and FAPbI_3_ PQDs but significantly reduces the density (and potentially the depth) of the electronic traps. Combined, these observations highlight the importance of the A-site cation to the electronic properties of the PQD film and point to control of the PQD composition and surface chemistry in order to enable high-performance optoelectronic devices such as solar cells.

## Methods and Experimental Details

### Materials

Cesium carbonate (Cs_2_CO_3_; 99.9%), oleic acid (OA; technical grade, 90%), 1-octadecene (1-ODE; technical grade, 90%), oleylamine (OLA; technical grade, 70%), methyl acetate (MeOAc; anhydrous, 99.5%), hexanes (reagent grade, ≥95%), octane (anhydrous, ≥99%), formamidinium acetate (FA-acetate; 99%), toluene (anhydrous, 99.8%), titanium diisopropoxide bis(acetylacetonate) (TAA; 75 wt % in isopropanol), 1-butanol (anhydrous, 99.8%), acetone (for HPLC, ≥99.9%), chlorobenzene (anhydrous, 99.8%), 4-*tert*-butylpyridine (*t*BP; 98%), and acetonitrile (anhydrous, 99.8%) were purchased from Sigma-Aldrich. Bis(trifluoromethane)sulfonimide lithium salt (Li-TFSI; 98+%) and lead iodide (PbI_2_, 99.9985%) were purchased from Alfa Aesar. Isopropanol (IPA; ACS Plus) was purchased from Fisher Chemical. Spiro-OMeTAD was purchased from Lumtec.

### CsPbI_3_ QD Synthesis

CsPbI_3_ QDs were prepared using a previously reported procedure.^2^ First, a solution of cesium oleate was prepared. 407 mg of Cs_2_CO_3_, 1.25 mL of OA, and 20 mL of 1-ODE were loaded into a 50 mL three-neck round-bottom flask with a stir bar and thermocouple. The solution was degassed under vacuum at 50 °C for 30 min before the flask was filled with nitrogen and the temperature was raised to 150 °C. After all the Cs_2_CO_3_ had dissolved, the temperature was reduced to 120 °C and maintained at that temperature for injection.

In a separate 50 mL round-bottom flask, 500 mg of PbI_2_ and 25 mL of 1-ODE were degassed at 120 °C under vacuum with stirring for 30 min. A mixture of 2.5 mL of OA and 2.5 mL of OLA was vortexed vigorously and heated to 130 °C for approximately 20 min on a hot plate. The OA/OLA mixture was injected into the reaction flask under vacuum, and the solution was degassed until the PbI_2_ fully dissolved to form a clear yellow solution. Then the flask was filled with nitrogen, and the solution was heated to 178 °C. At 178 °C, 2 mL of the Cs-oleate solution was swiftly injected, and the reaction was immediately quenched in an ice–water bath.

To isolate the CsPbI_3_ QDs from excess precursors, the reaction mixture was separated into two 50 mL centrifuge tubes, and 70 mL of MeOAc was added before centrifugation at 7500 rpm (7798*g*) for 5 min. The supernatant was decanted, and the precipitated QDs were redispersed in 5 mL of hexane and consolidated in a single centrifuge tube. The QDs were reprecipitated by the addition of 5 mL of MeOAc and centrifuged again at 7500 rpm (7798*g*) for 5 min. The bright red supernatant was decanted, and the product QDs (solid) were redispersed in 10 mL hexanes. These QDs were stored in a refrigerator overnight. The solution was centrifuged at 7500 rpm (7798*g*), discarding the precipitate. The product QDs were concentrated by evaporating the hexane in the supernatant and finally redispersing the QDs in 1–2 mL of octane.

### FAPbI_3_ QD Synthesis

FAPbI_3_ QDs were prepared using a previously reported procedure.^2^ A solution of FA-oleate was prepared. 573.1 mg of FA-acetate, 11 mL of OA, and 20 mL of 1-ODE were loaded into a 50 mL three-neck round-bottom flask with a stir bar and thermocouple. The solution was degassed under vacuum at 50 °C for 30 min before the flask was filled with nitrogen, and the temperature was raised to 120 °C. After all the FA-acetate had dissolved, the temperature was reduced to 80 °C and maintained at that temperature for injection.

In a separate 50 mL round-bottom flask, 344 mg of PbI_2_ and 20 mL of 1-ODE were degassed at 120 °C under vacuum with stirring for 30 min. A mixture of 4 mL of OA and 2 mL of OLA was vortexed vigorously and heated to 120 °C for approximately 20 min on a hot plate. The OA/OLA mixture was injected into the reaction flask under vacuum, and the solution was degassed until the PbI_2_ fully dissolved to form a clear yellow solution. Then the flask was filled with nitrogen, and the solution was cooled to 80 °C. At 80 °C, 5 mL of the FA-oleate solution was swiftly injected, and after 5 s the reaction was quenched in an ice–water bath.

To isolate the FAPbI_3_ QDs from excess precursors, 1 mL of toluene and 5 mL of MeOAc were added to the reaction mixture, and the solution was centrifuged at 8000 rpm (8873*g*) for 10 min. The supernatant was decanted, and the resulting QD precipitate was dispersed in 7 mL of toluene, reprecipitated with 5 mL of MeOAc, and centrifuged at 8000 rpm (8873*g*) for 10 min. After decanting the supernatant, the product QDs (solid) were dispersed in 5–7 mL of octane and stored under nitrogen.

### Cs_0.5_FA_0.5_PbI_3_ QD Synthesis

The colloidal solutions of CsPbI_3_ and FAPbI_3_ QDs were mixed in approximately equimolar quantities to produce the Cs_0.5_FA_0.5_PbI_3_ QDs. The mixture was reacted at 45 °C overnight. Photoluminescence was used to monitor the extent of the reaction and final stoichiometry.

### QD Film Preparation

Quantum dot films were prepared by spin deposition in a nitrogen flowbox with 20 ± 2% relative humidity. A concentrated QD solution in octane (approximately 60 mg/mL) solution was used. After filtering the QD solution through a 0.2 μm syringe filter, 10 μL of the QD solution was deposited dynamically at 2000 rpm for 30 s.

For films with ligand exchange, a solution of Pb(NO_3_)_2_ in MeOAc was prepared by sonicating 40 mg of Pb(NO_3_)_2_ in 40 mL of MeOAc for 30 min. The solution was carefully decanted to remove undissolved Pb(NO_3_)_2_. Immediately after spin deposition, the QD film was dipped in the Pb(NO_3_)_2_/MeOAc solution for ∼3 s, followed by neat MeOAc for ∼3 s.

### Solar Cell Device Fabrication

Prepatterned ITO-coated glass substrates were cleaned by successively sonicating for 15 min in acetone followed by 15 min in IPA. The substrates were then cleaned with UV-ozone for 15 min. TiO_2_ was deposited on the ITO-coated glass substrates by spin-casting a solution of 150 μL of TAA in 1.85 mL of 1-butanol using the following spin recipe: 700 rpm for 10 s, 1000 rpm for 10 s, and 2000 rpm for 30 s. The resulting film was dried for 5 min at 125 °C on a hot plate, followed by annealing at 450 °C for 1 h. The TiO_2_ surface was cleaned by UV-ozone for 15 min prior to QD deposition. QDs were deposited as previously described with ligand exchange, repeating the process four times to build up a thick QD film. A spiro-OMeTAD hole transport layer was spin-cast at 5000 rpm for 30 s from a solution of 72 mg of spiro-OMeTAD, 1 mL of chlorobenzene, 28.8 μL of *t*BP, and 18 μL of Li-TFSI solution (520 mg/mL Li-TFSI in acetonitrile). A 15 nm layer of MoO_*x*_ was thermally evaporated at a rate of 0.1–0.5 Å/s, and then a 100 nm Al layer was thermally evaporated at a rate of 0.5–2.0 Å/s.

### Extinction Spectra

Extinction spectra were recorded using a commercial spectrometer with an integration sphere. Films were held inside the center of the sphere in order to account for scattering and reflection.

### Photoluminescence Spectra

Photoluminescence spectra were measured using a commercial fluorimeter. Spectra in Figure S2 were recorded using 500 nm excitation and emission collected by a photomultiplier tube from 600 to 830 nm. PLQY measurements were collected using an integrating sphere and a liquid nitrogen-cooled CCD collecting from 400 to 900 nm. Correction factors were created before measuring the PLQY using a calibrated LED light source to correct for wavelength dependence of the sphere, fiber optics, monochromator, detector, and other optics. PLQY measurements were measured using 500 nm excitation light from a laser-driven white light source fitted with a monochromator.

### Absolute Photoluminescence Hyperspectral Imaging

The PL image detection was performed with a CCD camera coupled to a liquid crystal tunable filter. The samples were excited with 455 nm broad illumination with a photon flux of ∼1.4 × 10^21^ photons·m^–2^ s^–1^ which is equivalent to 1 sun conditions for a material with a band gap of ∼1.746 eV.

The QFLS maps were calculated with Δ*E*_f_ = Δ*E*_f_^rad^ + *kT* ln{*Q*_e_^PL^}, where Δ*E*_f_^rad^ is the QFLS at the radiative limit and *Q*_e_^PL^ is the external PLQY.^66^ The radiative limit of the QFLS, Δ*E*_f_^rad^ was calculated from the external quantum efficiency (EQE) spectrum of a representative CsPbI_3_ QDs solar cell with the method reported by Krückemeier *et al*.^67,68^ The analysis yielded a radiative QFLS, Δ*E*_f_^rad^ ≈ 1.46 eV. The global systematic error is approximately 20 meV, which corresponds to a factor of 2 in the *Q*_e_^PL^.

### TRPL Measurement

The TRPL was measured using time-correlated single photon counting with a Si avalanche photodiode after excitation with a 470 nm, 300 fs laser pulse with a 1.1 MHz repetition rate. The TRPL decay data (*i*.*e*., Figure 3a) were measured until 2000 counts were reached in one channel. Integrated TRPL data (*i*.*e*., Figure 4) were normalized by dividing by the maximum value, which occurs at the highest excitation fluence.

## Supporting Information Available

Modeling methods and results, EQE data, *J*–*V* data, PLQY, kinetic parameters from TRPL modeling, TRPL data for FA-containing QDs, and TRPL of CsPbI_3_ PQDs before ligand exchange (PDF)
