# Supplementary material for: Probing the Origin of the Open Circuit Voltage in Perovskite Quantum Dot Photovoltaics
Source: ACS Nano. 2021 Dec 3;15(12):19334–44. doi: 10.1021/acsnano.1c05642 (PMC10156082; doi:10.1021/acsnano.1c05642)
Supplement: Supplementary file 1 — nn1c05642_si_001.pdf [file nn1c05642_si_001.pdf]

Supporting Information for

Probing the Origin of the Open Circuit Voltage in Perovskite Quantum Dot Photovoltaics

Brian M. Wieliczka[1], José A. Márquez[2], Alexandra M. Bothwell[1], Qian Zhao[1], Taylor Moot[1], Kaitlyn T. VanSant[1], Andrew J. Ferguson[1], Thomas Unold*[,2], Darius Kuciauskas*[,1], Joseph M. Luther*[,1]

[1] *National Renewable Energy Laboratory, Golden, CO, 80401, USA*
[2] *Department of Structure and Dynamics of Energy Materials, Helmholtz-Zentrum-Berlin für Materialien und Energie GmbH, Hahn-Meitner Platz 1, 14109 Berlin, Germany*

* *Corresponding Authors: unold@helmholtz-berlin.de, darius.kuciauskas@nrel.gov, joey.luther@nrel.gov*

Table of Contents

S3 Figure S1. Representative JV curve

S4 Figure S2. Extinction and PL spectra of PQD films

S5 Table S1. Photoluminescence quantum yield and quasi-Fermi level splitting

S6 Figure S3. External quantum efficiency spectrum

S7 Table S2. Time resolved photoluminescence fitting parameters

S8 Kinetic model for TRPL data

S9 Figure S4. Kinetic simulation of injection-dependent TRPL decays

S10 Table S3. Kinetic parameters derived from TRPL simulations

S11 Figure S5. Normalized and integrated TRPL data for $CsPbI_3$ before ligand exchange

S13 Kinetic model for high-injection TRPL data

S14 Figure S6. Kinetic scheme used in simulations of high injection TRPL data

S15 Figure S7. Simulation of free charge carrier-dependent TRPL decays

S16 Figure S8. Time resolved photoluminescence data for $Cs_{0.5}FA_{0.5}PbI_3$ and $FAPbI_3$

S17 Figure S9. PLQY of $CsPbI_3$, $Cs_{0.5}FA_{0.5}PbI_3$, and $FAPbI_3$ PQDs

S18 References

Figure S1 JV curve of representative $CsPbI_3$ PQD solar cell.

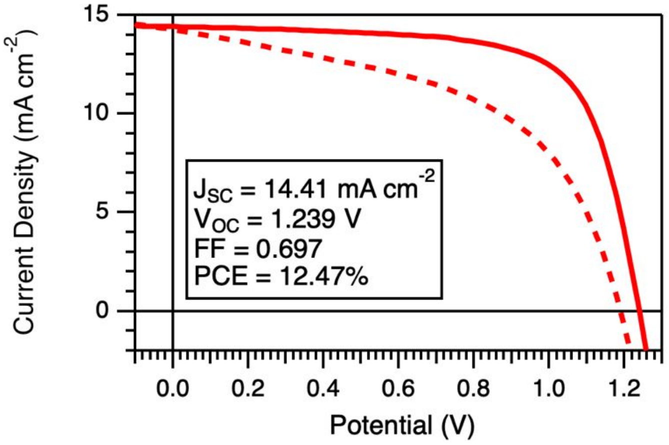

Figure S2. Comparison of extinction and photoluminescence spectra for $CsPbI_3$ (a), $Cs_{0.5}FA_{0.5}PbI_3$ (b), and $FAPbI_3$ (c) for various processing stages: 1-layer without ligand exchange (black), 1-layer with ligand exchange (red), and 4-layers with ligand exchange (blue). PL spectra were normalized by dividing by the maximum value.

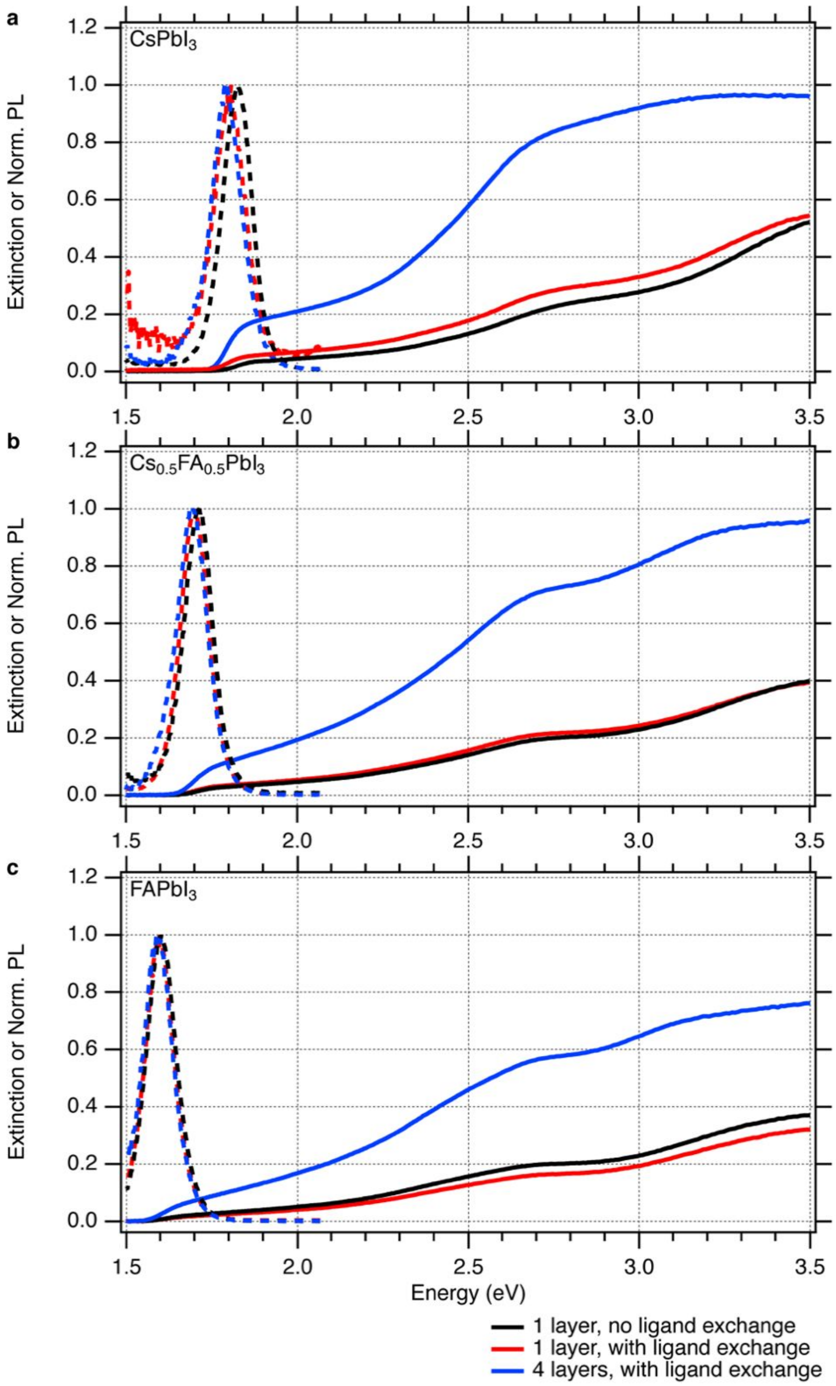

Table S1. Photoluminescence quantum yield and quasi-Fermi level splitting of $CsPbI_3$ PQD films and $CsPbI_3$ PQD solar cells. The method for collecting the data is noted for each sample.

**Sample**	**PLQY (%)**	**QFLS (eV)**	**Method**
Device-Ready QDs Solution	57.0 ± 5.1	1.45 ± 0.03	PLQY using Integrating Sphere
Glass/$CsPbI_3$ QDs before ligand exchange	5.3 ± 0.3	1.383 ± 0.002	PLQY using Integrating Sphere
Glass/$CsPbI_3$ QDs	1.80×10^{-2}	1.24 ± 0.02	Hyperspectral Imaging
ITO/TiO_2/$CsPbI_3$/Spiro/MoO_x/Al (Devices)	2.00×10^{-2}	1.24 ± 0.02	Hyperspectral Imaging
ITO/TiO_2/$CsPbI_3$/Spiro/MoO_x/Al (Encapsulated Devices)	2.00×10^{-2}	1.24 ± 0.02	Hyperspectral Imaging

Figure S3. External quantum efficiency spectrum used to determine the open circuit voltage at the radiative limit (V_{oc}^{rad}) with the calculated electroluminescence (EL) spectrum of the $CsPbI_3$ PQDs solar cells. The EQE spectrum was extended to lower photon energies by extrapolating the EQE with a 14 meV Urbach tail using the methodology previously reported.[1]

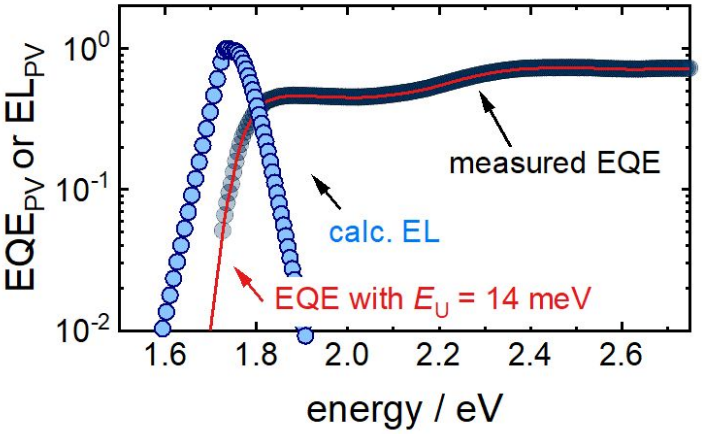

Table S2. Time-resolved photoluminescence fitting parameters.

**Excitation fluence (photons/cm^2 pulse)**	**A_1 decay component (%)**	**Lifetime t_1 (ns)**	**A_2 decay component (%)**	**Lifetime t_2 (ns)**
9.1×10^{9}	92	0.49	8	9.8
4.6×10^{10}	85	0.48	15	5.1
9.1×10^{10}	80	0.52	20	4.4
1.9×10^{11}	75	0.58	25	4
3.8×10^{11}	70	0.67	30	3.5
7.6×10^{11}	65	0.82	35	3.4
1.5×10^{12}	65	1	35	2.8
3.0×10^{12}	63	1.1	37	2.2
4.6×10^{12}	80	1.1	20	2.2

Kinetic model for low-injection TRPL data

Kinetic model for TRPL data in the low-injection regime (from Ref. 2):

$$\frac{dn}{dt} = -k_1 n - k_2 n(N_t - n_t) + k_3 n_t \quad \text{(S1)}$$

$$\frac{dn_t}{dt} = -k_3 n_t + k_2 n(N_t - n_t) - k_4 n_t \quad \text{(S2)}$$

Where n/n_t are electron densities in conduction band/trap state, N_t is trap density, $k_1/k_2/k_3/k_4$ are rate constants shown in Figure 3b. Following Ref. 2, for simplicity we did not consider hole trapping. Detrapping rate constant k_3 exponentially depends on the activation energy E_a of the electron trap

$$k_3 = n_t \sigma_n v_{th} N_C exp\left(-\frac{E_a}{k_B T}\right) \quad \text{(S3)}$$

Where σ_n is cross-section, v_{th} is thermal velocity, N_C is density of states in the conduction band, k_B is Boltzmann's constant, and T is temperature. PL intensity is calculated as a product of free electrons and free holes (ref. 2).

By modeling TRPL data using Eqs. (S1-S3), we determine E_a, N_t, and $(k_1)^{-1}$ (see Table S3). A more detailed TRPL model including radiative recombination is described below. Model of Eqs. (S1) – (S3) has a smaller number of adjustable parameters and is easier to apply to the low injection data. Modeling is not very sensitive to rates k_2 and k_4 and assumed values for these parameters are $k_2^{-1} = 1$ ns, and $k_4^{-1} = 100$ ns. Simulated kinetic decays for $CsPbI_3$ PQDs are shown in Figure S1, and parameters used in kinetic simulations for $CsPbI_3$, $Cs_{0.5}FA_{0.5}PbI_3$, and $FAPbI_3$ are summarized in Table S3.

Figure S4. Kinetic simulation of injection-dependent TRPL decays for $CsPbI_3$ PQDs. Experimental data is given in Figure 3a. Black solid line shows the lowest injection where photogenerated carrier density is lower than trap density. Purple and grey lines correspond to injection where photogenerated carrier density exceeds trap density.

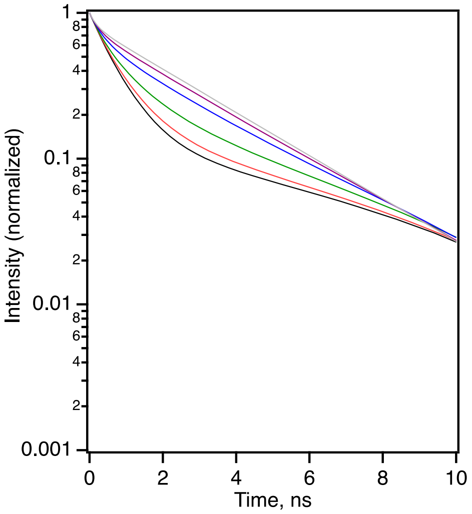

Table S3. Defect-mediated recombination lifetimes, trap densities, and trap activation energies. Kinetic parameters in used to simulate injection dependence for time integrated TRPL data, shown as solid lines in Fig. 3(b), Fig. 4(c), and Fig. 4(d).

	$CsPbI_3$	$Cs_{0.5}FA_{0.5}PbI_3$	$FAPbI_3$
k_1^{-1} (ns)	3	7	40
N_t (cm^{-3})	3×10^{16}	8×10^{15}	8×10^{14}
E_a (mV)	200	150	70

Figure S5. TRPL data for $CsPbI_3$ PQD films before ligand exchange with varying excitation photon fluence (photons/(pulse·cm^2)) indicated in the legend (a). Normalized integrated photoluminescence intensity (data points) *versus* excitation fluence with model results (lines) for $CsPbI_3$ before and after ligand exchange, black and green, respectively (b).

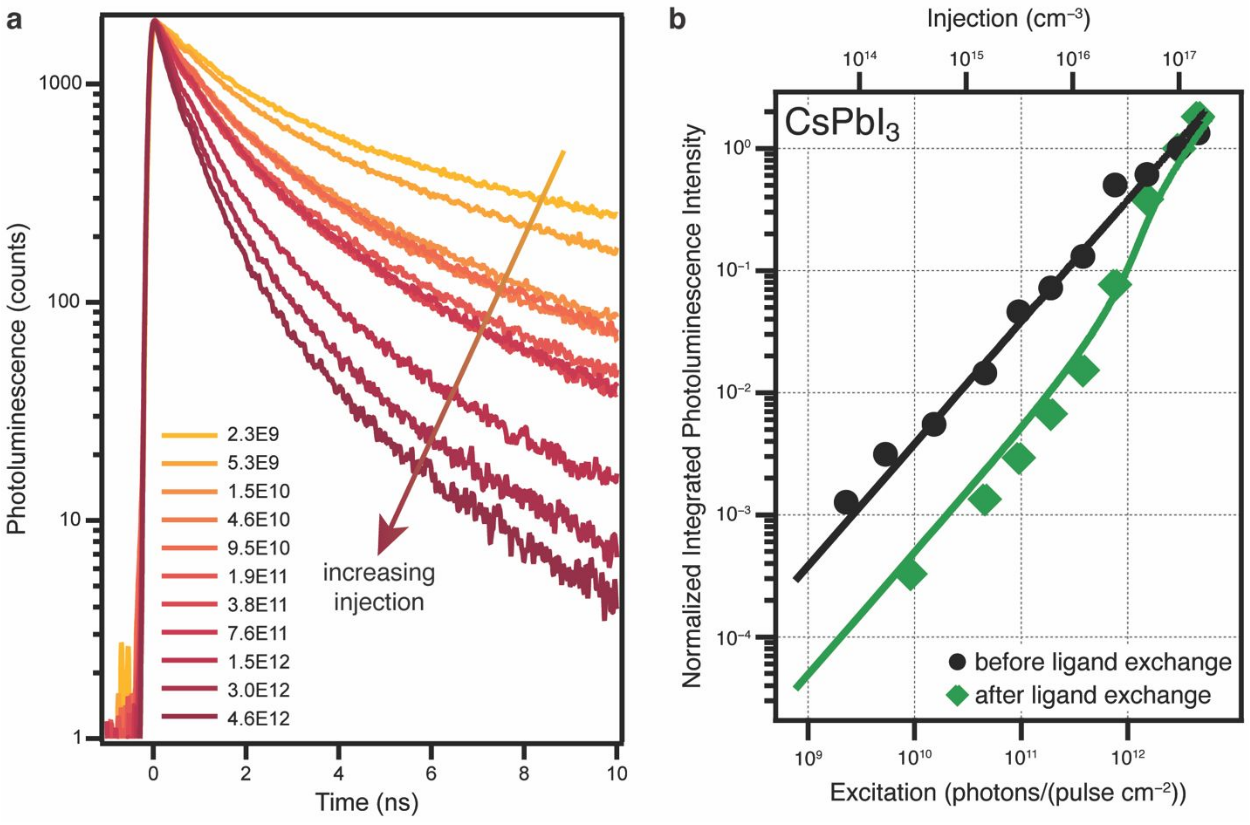

Representative TRPL data for $CsPbI_3$ PDQ films before ligand exchange (Figure Sxa). These samples have lower background carrier density (doping) and as a result, the TRPL data is dominated by radiative recombination in the experimentally accessible injection range. This is evident by the fact that the highest lifetime was obtained with the lowest excitation fluence, and lifetimes decrease when injection is increased. Please note the very different excitation fluence dependence for solid-state ligand exchanged samples (compared to Figure 3a in the manuscript).

Time-integrated TRPL data (Figure Sxb, also shown below) does not indicate the characteristic transition from empty to filled trap states (black *versus* green). Therefore, prior to solid state ligand exchange, we are not able to quantify either defect-mediated recombination lifetime, trap density, or background carrier density (doping).

Such samples have very different recombination properties, which is best illustrated by by their ~300 times higher radiative efficiency (Table S1). This TRPL data is in agreement with this result.

Kinetic model for high-injection TRPL data

As described in earlier section, modeling minority carrier dynamics (from low injection TRPL data) established bulk carrier lifetime, trap density and activation energy (Table S3). To analyze radiative recombination from high injection TRPL data both electron and hole dynamics need to be considered. We used a previously published MATLAB script[3] to solve time-dependent transport equations for electrons n (Eq. (S4)) and holes p (Eq. (S5)):

$$\frac{\partial n}{\partial t} = k_B T \mu \frac{\partial^2 n}{\partial z^2} + \sum_{i=1}^{2} (e_{e,i} - e_{c,i}) - R_{rad} + G \qquad \text{(S4)}$$

$$\frac{\partial p}{\partial t} = k_B T \mu \frac{\partial^2 n}{\partial z^2} + \sum_{i=1}^{2} (h_{e,i} - h_{c,i}) - R_{rad} + G \qquad \text{(S5)}$$

$$e_{e,i} = n_{t,i} \sigma_{n,i} v_{th} N_C exp\left(- \frac{E_{t,i}}{k_B T} \right) \qquad \text{(S6)}$$

$$e_{c,i} = n(N_{t,i} - n_{t,i}) \sigma_{n,i} v_{th} \qquad \text{(S7)}$$

$$h_{e,i} = (N_{t,i} - n_{t,i}) \sigma_{p,i} v_{th} N_V exp\left(- \frac{E_g - E_{t,i}}{k_B T} \right) \qquad \text{(S8)}$$

$$h_{c,i} = p n_{t,i} \sigma_{p,i} v_{th} \qquad \text{(S9)}$$

Where Eqs. (S6) – (S9) describe electron/hole capture and emission rates to and from the defect levels i as illustrated in Fig. S5. Two defect levels (i = 1, 2) were considered: midgap defect-mediated recombination centers (which set the value of minority carrier lifetime in the absence of traps) and more shallow "traps" with activation energy $E_{t,2}$, density $N_{t,2,}$ and trapped electron density $n_{t,2}$. E_g is bandgap, N_C and N_V are densities of states for conduction and valence bands, v_{th} is thermal velocity, and $\sigma_{n,i}/\sigma_{h,i}$ are electron/hole capture cross-sections for each defect level, k_B is Boltzmann's constant, and T is temperature. Mobility m = 20 $cm^2/(Vs)$ was assumed to be the same for electrons and holes. Surface recombination was included with boundary conditions, and surface recombination velocity was assumed to be low, $S_{front}=S_{back}=1$ cm/s. Low value of surface recombination velocity is justified as described in the main text. Generation rate (G in Eqs. (1)-

(2)) was approximated by a Gaussian laser pulse with 300 fs width and Beer's law when absorber thickness was d = 300 nm and absorption coefficient 1×10^5 cm^{-1}.

Figure S6. Kinetic scheme used in simulations of high injection TRPL data.

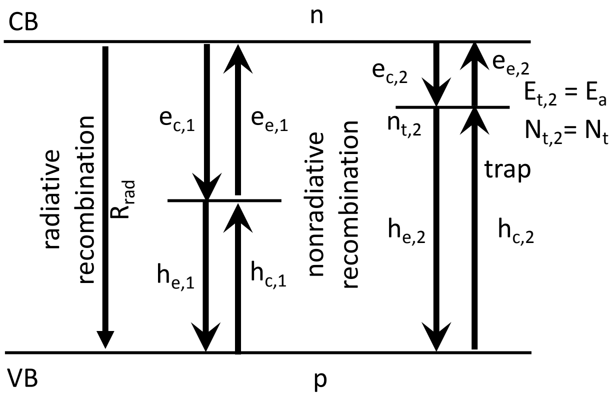

Time-dependent photoluminescence intensity $I_{PL}(t)$ was calculated by integrating radiative recombination rate R_{rad} over the thickness of the absorber

$$I_{PL}(t) = B\int_0^d \left(n(z,t)p(z,t) - n_i^2\right)dz \qquad \text{(S10)}$$

Where n_i is intrinsic carrier density, $n_i = \sqrt{N_C N_V}\exp\left(-\frac{E_g}{2k_BT}\right)$. We assumed radiative recombination coefficient B = 1.67×10^{-10} cm^3/s, corresponding to values given in recent review.[4]

Figure S6 shows TRPL decays for $CsPbI_3$, (Cs,FA)PbI_3, and $FAPbI_3$ PQD films when excitation fluence was 4.4×10^{12} photons/(cm^{2}pulse). Simulations were used to estimate hole density p in the absorber. In modeling, we did not adjust trap parameters and bulk lifetimes determined as described earlier. While bulk carrier lifetime, trap density and energy differ between PQD films, all data is consistent with p = (1 - 3.3) $\times10^{18}$ cm^{-3}, which indicates high hole density in the absorber.

Figure S7. TRPL decays for $CsPbI_3$ (a), $Cs_{0.5}FA_{0.5}PbI_3$ (b), and $FAPbI_3$ (c) PQD films when excitation fluence was 4.4×10^{12} photons/(cm^2pulse). Simulation with Eqs. (S4) – (S10) using parameters in Table S3 and varied hole density in the absorber, as indicated in the legends.

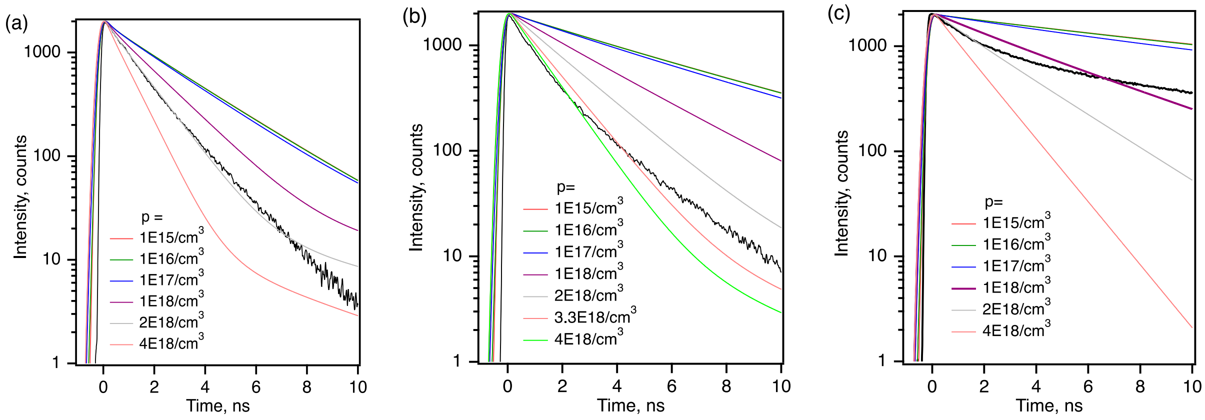

Figure S8. TRPL data for $Cs_{0.5}FA_{0.5}PbI_3$ (a) and $FAPbI_3$ (b) PQD films after ligand exchange with varying 470 nm excitation photon fluence (photons/(pulse·cm^2)) indicated in the legend.

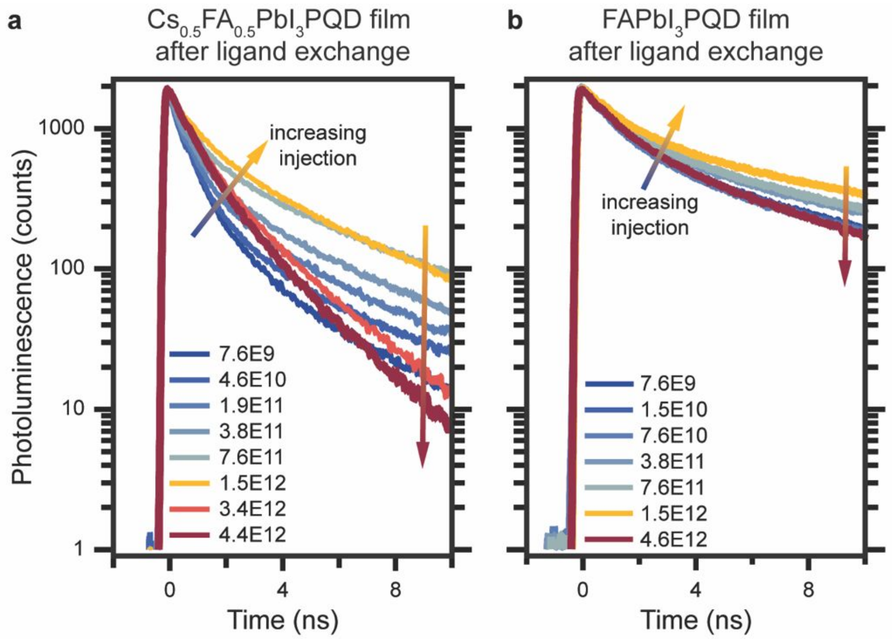

Figure S9. Photoluminescence quantum yield of $CsPbI_3$, $Cs_{0.5}FA_{0.5}PbI_3$, and $FAPbI_3$ PQD solutions and 1-layer thick films before and after ligand exchange.

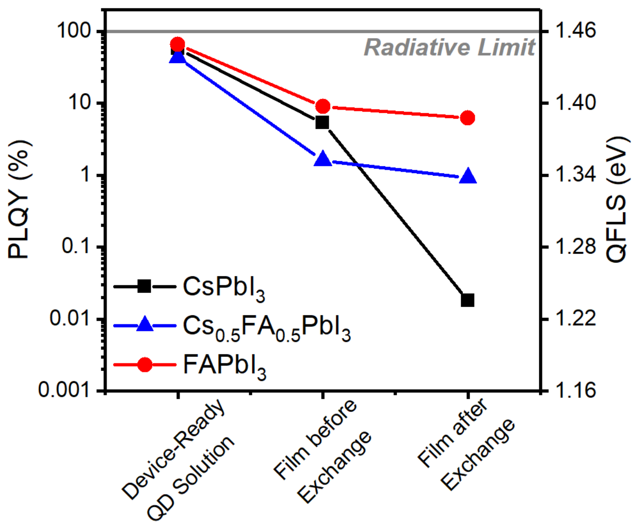

References

1. Krückemeier, L.; Rau, U.; Stolterfoht, M.; Kirchartz, T., How to Report Record Open-Circuit Voltages in Lead-Halide Perovskite Solar Cells. *Advanced Energy Materials* **2020,** *10*, 1902573.

2. Yamada, Y.; Yamada, T.; Shimazaki, A.; Wakamiya, A.; Kanemitsu, Y., Interfacial Charge-Carrier Trapping in $CH_3NH_3PbI_3$-Based Heterolayered Structures Revealed by Time-Resolved Photoluminescence Spectroscopy. *J Phys Chem Lett* **2016,** *7*, 1972-7.

3. Weiss, T. P.; Bissig, B.; Feurer, T.; Carron, R.; Buecheler, S.; Tiwari, A. N., Bulk and Surface Recombination Properties in Thin Film Semiconductors with Different Surface Treatments from Time-Resolved Photoluminescence Measurements. *Sci Rep* **2019,** *9*, 5385.

4. Kirchartz, T.; Márquez, J. A.; Stolterfoht, M.; Unold, T., Photoluminescence-Based Characterization of Halide Perovskites for Photovoltaics. *Advanced Energy Materials* **2020,** *10*, 1904134.
